# Analysis of pathogen distribution and serum procalcitonin level alterations in patients with type 2 diabetes mellitus complicated by urinary tract infections

**DOI:** 10.3389/fendo.2025.1609966

**Published:** 2025-05-29

**Authors:** Yuan Pu, Yirong Teng, Yinghua Li, Yunchun Zhou, Ming Gao, Xianglan Chen, Zilin Yan, Xia Li, Rong Wei, Zhaowei Teng

**Affiliations:** ^1^ Department of General Medicine, The Sixth Affiliated Hospital of Kunming Medical University, Yuxi, China; ^2^ Department of Scientific Research Management, The Sixth Affiliated Hospital of Kunming Medical University, Yuxi, China; ^3^ Department of Endocrinology and Metabolism, The Sixth Affiliated Hospital of Kunming Medical University, Yuxi, China; ^4^ Department of Center Laboratory, The Second Affiliated Hospital of Kunming Medical University, Kunming, China

**Keywords:** type 2 diabetes, urinary tract infection, pathogens, antibiotic resistance, procalcitonin

## Abstract

**Objective:**

To investigate the distribution and antibiotic resistance of pathogens in patients with Type 2 Diabetes Mellitus (T2DM) complicated by Urinary Tract Infections (UTIs), and to explore the value of serum Procalcitonin (PCT) level alterations in the diagnosis and assessment of disease severity in these patients.

**Methods:**

This retrospective analysis included 100 patients with T2DM complicated by UTIs admitted to the Sixth Affiliated Hospital of Kunming Medical University between January 2021 and August 2024, who constituted the Infection group. A control group, the T2DM group, consisted of 100 patients with T2DM without UTIs. Key demographic data were retrospectively analyzed and evaluated. Bacterial isolation and identification, along with antimicrobial susceptibility testing using an automated analyzer, were performed to determine the distribution and antimicrobial resistance profiles of the isolated pathogens. Serum PCT levels were measured using electrochemiluminescence immunoassay. Receiver Operating Characteristic (ROC) curve analysis was used to evaluate the diagnostic value of PCT for T2DM complicated by UTIs. The optimal cutoff value was calculated using the Youden index derived from the ROC curve.

**Results:**

146 pathogenic strains were isolated from the 200 submitted specimens. Gram-negative bacteria accounted for 66.44% (n=97), Gram-positive bacteria for 14.38% (n=21), and fungi for 19.18% (n=28). *Escherichia coli* was the most common pathogen. Antimicrobial susceptibility testing revealed high resistance rates of *E. coli* to levofloxacin and ampicillin, while no resistance was observed to amikacin and tigecycline. *Candida glabrata* exhibited high resistance to itraconazole, but no resistance to 5-flucytosine, fluconazole, or voriconazole. Serum PCT levels were significantly elevated in the infection group compared to the T2DM group (P< 0.05). ROC curve analysis revealed that the area under the curve (AUC) for PCT in diagnosing T2DM complicated by UTIs was 0.700 (95% CI, 0.628-0.772). The maximum Youden index was calculated to be 0.36, corresponding to an optimal cutoff value of 0.0965 ng/L on the ROC curve.

**Conclusion:**

*Escherichia coli* and *Candida glabrata* are the predominant pathogens in T2DM patients with UTIs. Serum PCT levels have moderate value in the diagnosis of patients with T2DM complicated by UTIs.

## 1 Introduction

Diabetes Mellitus (DM) is a common endocrine metabolic disorder primarily characterized by insulin resistance, impaired insulin secretion, and abnormally elevated hepatic glycogen production (1). Globally, approximately 537 million individuals have diabetes, with Type 2 Diabetes Mellitus (T2DM) accounting for about 90% of cases (2). With the accelerating aging of the population and changes in lifestyle in China, the prevalence of diabetes among residents continues to rise; the prevalence among individuals aged ≥18 years reached 12.8% in 2017 (3). Statistical data estimate that the global number of diabetes patients will reach approximately 1.31 billion by 2050 (4). The mechanisms contributing to Urinary Tract Infections (UTIs) in patients with diabetes primarily involve Diabetic Cystopathy, diabetes-induced immune dysfunction, enhanced adhesion of bacteria to uroepithelial cells, and the glucose content in urine (5). The immune function of diabetic patients is often compromised, leading to an increased risk of infection. Studies have shown that immune cell function is weakened in patients with T2DM, particularly the reduced activity of macrophages and lymphocytes, which significantly diminishes their resistance to infection (6). High glucose concentrations in urine provide an ideal growth environment for bacteria, thereby significantly increasing the risk of UTIs (7). UTIs, as one of the common complications of T2DM, have become a significant factor affecting patients’ quality of life. A systematic review and meta-analysis showed that the overall incidence of UTIs in patients with T2DM is 11.5%, with an incidence of 14.2% in female patients and 6.1% in male patients (8).

Although antimicrobial agents can effectively reduce the incidence and mortality of UTIs, the causative pathogens of UTIs have increasingly exhibited antibiotic resistance in recent years (9). The differences in antibiotic resistance patterns of pathogens isolated from diabetic patients with UTIs compared to non-diabetic patients remain controversial (5). Therefore, clarifying the distribution characteristics and antibiotic resistance status of pathogens in patients with T2DM complicated by UTIs is an urgent and important issue.

Furthermore, serum Procalcitonin (PCT), a widely used biomarker for infection diagnosis, has garnered increasing attention for its clinical value in patients with T2DM (10). Existing studies have shown that PCT has high sensitivity and specificity in the diagnosis of UTIs in elderly patients with T2DM (11). Based on this, the present study aimed to analyze the pathogen distribution and antibiotic resistance characteristics in patients with T2DM complicated by UTIs, further screen for risk factors influencing the occurrence of UTIs, and explore the value of serum PCT level changes in the diagnosis and assessment of disease severity in this population. Through these analyses, we expect to provide new reference data for the prevention and treatment of T2DM complicated by UTIs.

## 2 Subjects and methods

### 2.1 Subjects

This retrospective case-control study investigated the Pathogen distribution and serum PCT levels in patients with T2DM complicated by UTIs. We selected 100 patients with T2DM complicated by UTIs admitted to the Sixth Affiliated Hospital of Kunming Medical University between January 2021 and August 2024 as the Infection group, and 100 patients with T2DM without UTIs as the T2DM group. Inclusion Criteria: Patients met the diagnostic criteria for T2DM according to the “Diagnosis and Classification of Diabetes: Standards of Care in Diabetes-2025” (12). Patients met the diagnostic criteria for UTIs according to the “Guidelines for the Prevention, Diagnosis, and Management of Urinary Tract Infections in Pediatrics and Adults: A WikiGuidelines Group Consensus Statement” (13). Exclusion Criteria: Patients with abnormal cardiac, hepatic, or renal function; pregnant or lactating women; patients with prostatitis or urinary obstruction; patients with other infections or urinary system lesions; patients with structural abnormalities of the urinary tract.

### 2.2 Methods

#### 2.2.1 Bacterial isolation and identification

Upon enrollment, morning urine samples were collected from patients using sterile disposable plastic cups. Before urine collection, the perineal area was cleaned with water, and the external genitalia were disinfected with povidone-iodine to reduce contamination by exogenous bacteria. Patients were instructed not to touch the inner wall of the container with their hands during collection to maintain sterility. The initial and final portions of urine were discarded, and a midstream urine sample was collected for testing. Urine samples were inoculated onto culture media. Columbia Blood Agar (CBA) was used for the culture of Gram-negative and Gram-positive bacteria, and Sabouraud Dextrose Agar (SDA) was used for the culture of fungi. Plates were incubated at 37°C in a constant temperature incubator for 24 hours for bacterial culture. After incubation, strains were isolated. Identification of isolates was performed using an automated bacterial identification system (VITEK MS mass spectrometer utilizing MALDI-TOF technology) strictly following the procedures specified in “Applications of MALDI-TOF mass spectrometry in clinical diagnostic microbiology” (14). A positive urine culture was defined as a colony count ≥ 10^5 CFU/mL.

#### 2.2.2 Antimicrobial susceptibility testing

Antimicrobial susceptibility testing was performed on all isolates using the VITEK 2 Compact automated system (utilizing multi-wavelength colorimetry and kinetic methods). Minimum inhibitory concentrations (MICs) were determined, and MIC distribution histograms were used for analysis. Barcoded test cards, including identification cards (GN, GP, VST, NH, and ANC) and antimicrobial susceptibility testing cards (AST-GN334, AST-GN335, AST-GP639, and GP68), were used. The Advanced Expert System (AES) was used to match and analyze the results.

#### 2.2.3 Serum PCT measurement

Serum PCT levels were measured using a commercially available procalcitonin assay kit based on electrochemiluminescence immunoassay. Five milliliters of fasting venous blood were collected from each subject in the morning. A sandwich immunoassay was employed. In the first incubation step, 18 μL of the sample was incubated with biotinylated monoclonal PCT antibody and ruthenium-labeled monoclonal PCT antibody, forming an antibody-antigen sandwich complex. In the second incubation step, streptavidin-coated magnetic microparticles were added and incubated, allowing the complex to bind to the magnetic beads through biotin-streptavidin interaction. The reaction mixture was then aspirated into the measuring cell, and the magnetic beads were captured on the electrode surface via magnetic force. Unbound substances were removed using ProCell II M. A defined voltage was applied to the electrode, inducing the chemiluminescence of the complex. The emitted light intensity was measured using a photomultiplier. The final PCT concentration was determined based on the instrument’s calibration curve.

### 2.3 Statistical analysis

This study employed a case-control design to compare the incidence of UTIs between the Infection group and the T2DM group. Based on previous literature, the overall incidence of UTIs in patients with T2DM is 11.5% (95% Confidence Interval: 7.8%-16.7%) (8). Using the sample size calculation formula for comparing proportions, assuming the infection rates in the Infection group and the T2DM group are *p*_1_ and *p*_2_ respectively, the sample size formula is:


$$n=\frac{{\left(Z_{\alpha /2}+Z_{\beta }\right)}^{2}\cdot \left[p_{1}\left(1-p_{1}\right)+p_{2}\left(1-p_{2}\right)\right]}{{\left(p_{1}-p_{2}\right)}^{2}}$$

Explanation of parameters: $Z_{\alpha /2}$ is the Z-value corresponding to the significance level α . For the commonly used significance level α = 0.05, the Z-value is 1.96. $Z_{\beta }$ is the Z-value corresponding to the statistical power β . Typically, a power of 80% is used, which corresponds to β = 0.2, and thus $Z_{\beta }$ = 0.84. $p_{1}$ and p2 represent the infection rates in the Infection group and the T2DM group, respectively. In this study, the incidence rate in the Infection group is 11.5% ( $p_{1}$ = 0.115), while it is assumed that there is no infection or the infection rate is low in the T2DM group, with $p_{2}$ set to 0. $p_{1}$ - $p_{2}$ represents the expected difference between the two groups. In this study, the difference is 0.115 - 0 = 0.115.

Based on this calculation, approximately 61 samples were needed per group to ensure that the difference in infection rates between the two groups could be effectively compared with a significance level of 0.05 and a power of 80%. This study included 100 samples in each of the Infection group and the T2DM group, totaling 200 samples, which met the statistical power requirements. Clinical data were retrieved and entered using the hospital’s medical record information system. Data entry was performed by two individuals independently, with a third individual verifying the data for quality control. Statistical analysis was performed using SPSS 29.0 software. Count data were expressed as numbers or percentages, and categorical variables were compared using the Chi-square test. Normally distributed measurement data were described using the mean and standard deviation (mean ± SD), and comparisons between the two groups were performed using the independent samples t-test (Corrected from paired t-test as per standard practice for independent groups). Non-normally distributed measurement data were described using the median and interquartile range M (P25, P75), and comparisons between the two groups were performed using a non-parametric test (Mann-Whitney U test). Key demographic characteristics were evaluated, and risk factors were analyzed using multivariate logistic regression with adjustment for confounding factors. ROC curve analysis was used to evaluate the diagnostic value of serum PCT for T2DM complicated by UTIs. The optimal cutoff value was calculated using the Youden index derived from the ROC curve. The Youden index = |Sensitivity - (1 - Specificity)| = |Sensitivity + Specificity - 1| = abs(Sensitivity - (1 - Specificity)). A larger index indicates a better screening test effect and higher authenticity. The value corresponding to the maximum Youden index is the cutoff value. A P-value< 0.05 was considered statistically significant. There were no missing data in this study. For non-normally distributed data (such as PCT), logarithmic transformation was used to effectively improve the data distribution characteristics and provide a better basis for subsequent statistical analysis.

## 3 Results

### 3.1 Baseline characteristics

A comparison of general characteristics between the groups, including gender, age, ethnicity, marital status, smoking, alcohol consumption, hospitalization days, duration of diabetes, presence of hypertension, Body Mass Index (BMI), fasting plasma glucose, HbA1c, and serum white blood cell count (WBC), showed statistically significant differences in gebder, hospitalization days, and WBC between the groups. There were no statistically significant differences in age, ethnicity, marital status, smoking, alcohol consumption, duration of diabetes, presence of hypertension, BMI, fasting plasma glucose, HbA1c (**Table 1**).

**Table 1:** Comparison of baseline characteristics of each group.

Variable		Infection group (n=100)	T2DM group (n=100)	Statistic	*P*-value
Gender (n)	Male	20	65	41.432	<0.001
	Female	80	35		
Age (years)		63.31 ± 16.06	58.41 ± 14.62	1.782	0.183
Ethnicity	Han Chinese	87	86	0.043	0.836
	Other	13	14		
Marital status	Married	96	94	0.421	0.516
	Unmarried	4	6		
Smoking	Yes	11	20	3.092	0.079
	No	89	80		
Alcohol consumption	Yes	14	24	3.249	0.071
	No	86	76		
Hospitalization days		9.70 ± 3.01	8.02 ± 2.08	7.555	0.007
duration of diabetes		8.58 ± 6.93	7.92 ± 7.86	3.859	0.051
Presence of hypertension	Yes	62	59	0.188	0.664
	No	38	41		
BMI(kg/m2)		24.92 ± 3.77	24.20 ± 4.05	2.795	0.096
FPG(mmol/L)		11.09 ± 3.55	9.22 ± 3.42	0.199	0.656
HbA1c (%)		10.09 ± 2.92	9.79 ± 2.43	0.415	0.520
WBC(10^9^/L)		8.80 ± 3.90	6.82 ± 1.57	3.769	<0.001

The data are expressed as the mean ± SD or number (%). T2DM, type 2 diabetes mellitus; BMI, body mass index; FPG, fasting plasma glucose; WBC, white blood cell.

### 3.2 Distribution of pathogens in T2DM patients with UTIs

**Figure 1:**
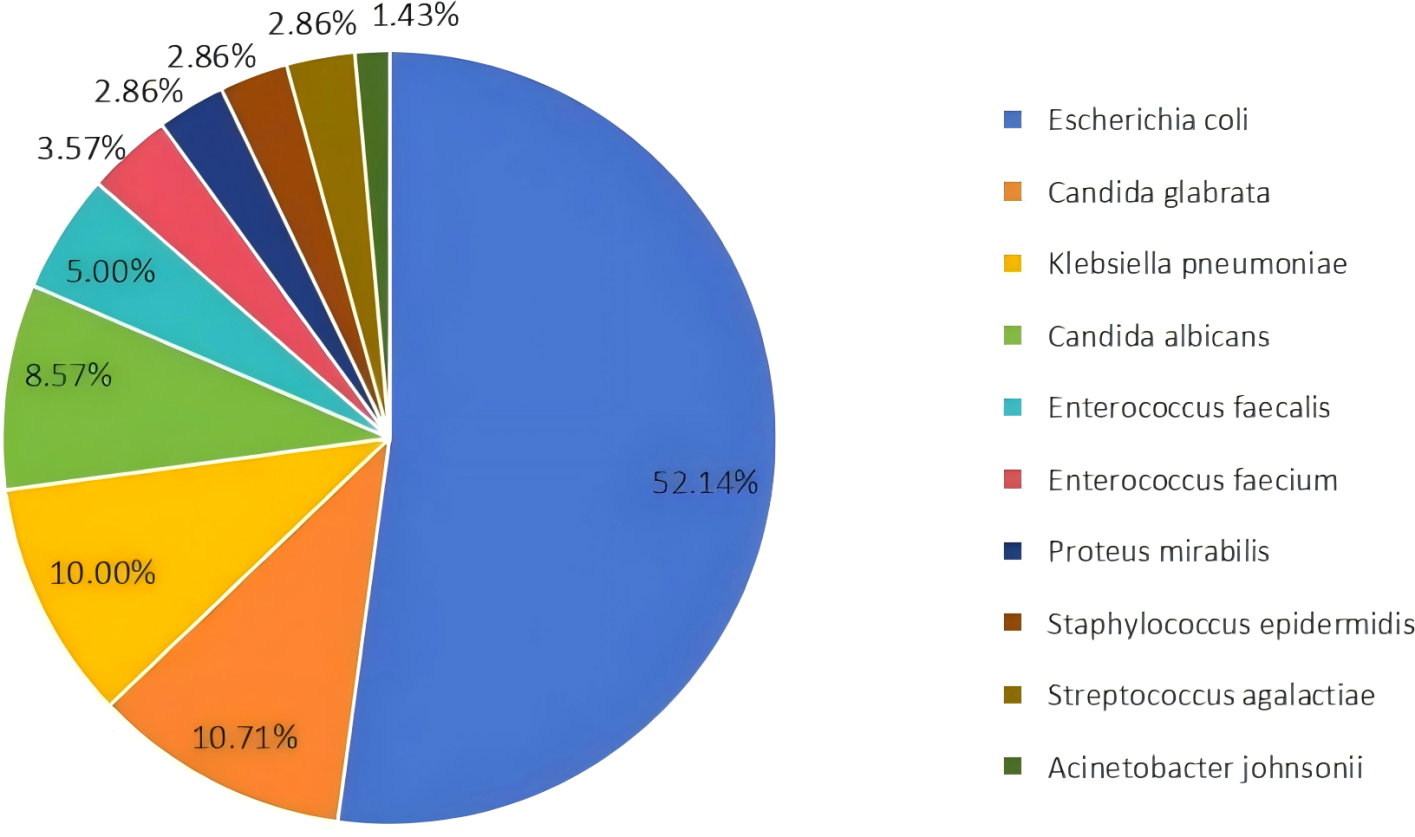
Distribution of the top ten bacterial species isolated from urine samples. *Escherichia coli* was the most prevalent species, accounting for 52.14% of the isolates.

Among the 100 submitted specimens, a total of 146 Pathogens were isolated, indicating that some patients had mixed infections (multiple pathogens isolated from a single specimen). The distribution of the top ten bacterial species isolated from urine specimens is shown in **Figure 1**. A total of 146 pathogenic strains were identified, with Gram-negative bacteria accounting for 66.44% (n=97), primarily *Escherichia coli* and *Klebsiella pneumoniae*. Gram-positive bacteria accounted for 14.38% (n=21), with *Enterococcus faecalis* being the most common (**Table 2**).

**Table 2:** Distribution and composition ratio of pathogens in patients with type 2 diabetes mellitus complicated with urinary tract infection.

Pathogen	Number of Strains (n=146)	Composition Ratio (%)
Gram-Negative Bacteria	97	66.44
*Escherichia coli*	73	50.00
*Klebsiella pneumoniae*	14	9.58
*Proteus mirabilis*	4	2.74
*Acinetobacter junii*	2	1.37
*Klebsiella oxytoca*	1	0.68
*Proteus vulgaris*	1	0.68
*Corynebacterium urealyticum*	1	0.68
*Morganella morganii*	1	0.68
Gram-Positive Bacteria	21	14.38
*Enterococcus faecalis*	7	4.79
*Enterococcus faecium*	5	2.54
*Staphylococcus epidermidis*	4	2.74
*Streptococcus agalactiae*	4	2.74
*Staphylococcus aureus*	1	0.68
Fungi	28	19.18
*Candida glabrata*	15	10.27
*Candida albicans*	12	8.22
*Candida krusei*	1	0.68

### 3.3 Antimicrobial resistance patterns of major pathogens

#### 3.3.1 Resistance rates of major gram-negative bacteria to commonly used antibiotics

*Escherichia coli* exhibited high resistance rates to ampicillin (86.3%) and levofloxacin (64.38%). No resistance was observed to amikacin or tigecycline. *Klebsiella pneumoniae* showed a high resistance rate to ampicillin (92.86%). No resistance was observed to amikacin, amoxicillin/clavulanate, tigecycline, or imipenem (**Table 3**).

**Table 3:** The resistance rate of major gram-negative bacteria to commonly used antibiotics.

Antibiotic	*Escherichia coli* (n=73)		*Klebsiella pneumoniae* (n=14)	
	Resistant Strains	Resistance Rate (%)	Resistant Strains	Resistance Rate (%)
Levofloxacin	47	64.38	2	14.29
Ampicillin	63	86.30	13	92.86
Amikacin	0	0.00	0	0.00
Gentamicin	20	27.40	2	14.29
Amoxicillin/Clavulanate	6	8.22	0	0.00
Tigecycline	0	0.00	0	0.00
Ceftazidime	19	26.03	2	14.29
Cefuroxime	37	50.68	4	28.57
Cefepime	25	34.25	2	14.29
Imipenem	3	4.11	0	0.00

#### 3.3.2 Resistance rates of major Gram-positive bacteria to commonly used antibiotics

*Enterococcus faecalis* exhibited high resistance rates to penicillin G, ampicillin, levofloxacin, and erythromycin (100% for each). No resistance was observed to vancomycin, teicoplanin, daptomycin, linezolid, or tigecycline (**Table 4**).

**Table 4:** The resistance rate of major gram-positive bacteria to commonly used antibiotics.

Antibiotic	*Enterococcus faecalis* (n=7)	
	Resistant Strains	Resistance Rate (%)
Penicillin G	7	100.00
Ampicillin	7	100.00
Vancomycin	0	0.00
Teicoplanin	0	0.00
Daptomycin	0	0.00
Levofloxacin	7	100.00
Erythromycin	7	100.00
Linezolid	0	0.00
High-Level Gentamicin	1	14.29
Tigecycline	0	0.00

### 3.4 Comparison of serum PCT levels between groups

Compared to the T2DM group, the infection group exhibited significantly elevated serum PCT levels (P< 0.05) (**Table 5**).

**Table 5:** Comparison of serum PCT levels among groups.

Indicator	Infection group	T2DM group	*Z*-value	*P*-value
PCT	0.09 (0.05,0.60)	0.05 (0.04,0.07)	-4.889	<0.001

PCT, procalcitonin.

### 3.5 ROC curve analysis of PCT for diagnosing UTIs in T2DM patients

**Figure 2:**
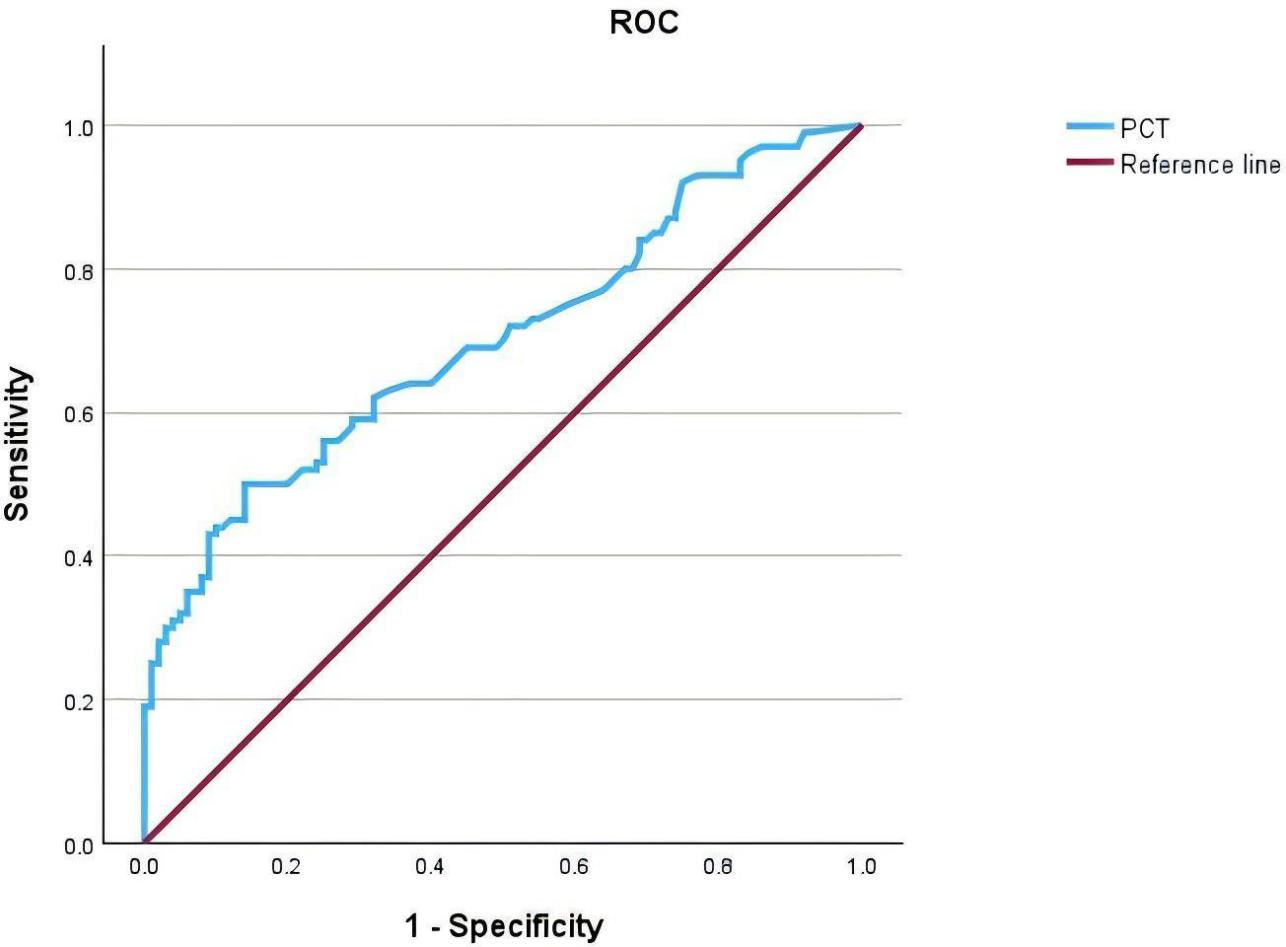
Receiver Operating Characteristic (ROC) curve of Procalcitonin (PCT) for the diagnosis of patients with type 2 diabetes mellitus (T2DM) complicated by urinary tract infections (UTIs). The area under the curve (AUC) is 0.700 (95% CI, 0.628-0.772), indicating fair diagnostic accuracy. The reference line represents the line of no discrimination.

ROC curve analysis showed that the area under the curve (AUC) for serum PCT in diagnosing UTIs in T2DM patients was 0.700 (95% CI, 0.628-0.772). The maximum Youden index was calculated to be 0.36, corresponding to an optimal cutoff value of 0.0965 ng/L on the ROC curve (**Figure 2**, **Table 6**).

**Table 6:** ROC curve analysis of the diagnostic value of PCT in type 2 diabetes mellitus complicated with urinary tract infection.

Item	Cutoff-Value	Area under curve	95%CI	Sensitivity (%)	Specificity (%)	Accuracy (%)
PCT	0.0965ng/L	0.700	0.628~0.772	50	86	68

ROC, receiver operating characteristic.

### 3.6 Analysis of risk factors for type 2 diabetes mellitus complicated by urinary tract infections

Multivariate logistic regression analysis was performed to identify independent risk factors for T2DM complicated by UTIs. Variables included in the model were those showing statistical significance in univariate analysis (gender, hospitalization days, WBC) as well as the clinically significant serum PCT level. The results showed that female, prolonged hospitalization, elevated WBC, and elevated PCT were independent risk factors for T2DM complicated by UTIs. These findings are presented in **Table 7**.

**Table 7:** Multivariable logistic regression study of risk factors for urinary tract infection in T2DM.

Risk factors	β	SE	Wald Value	OR	95%CI	P-value
Gender (ref = Female)	-2.053	0.382	28.932	0.128	0.061~0.271	<0.001
Hospitalization days	0.193	0.086	5.026	1.213	1.025~1.437	0.025
WBC	0.247	0.092	7.158	1.28	1.068~1.535	0.007
PCT	0.732	0.217	11.391	2.079	1.359~3.180	<0.001

WBC, white blood cell; PCT, procalcitonin.

## 4 Discussion

### 4.1 Pathogen distribution and antimicrobial resistance

Studies have shown that 40% of diabetic patients have varying degrees of bacteriuria, and the risk of death from UTIs in diabetic patients is more than 5 times higher than in non-diabetic patients (15). After T2DM patients develop UTIs, the progression of the disease will be accelerated, and the recurrence rate is relatively high. After multiple infections and treatments, the drug resistance of pathogens may be enhanced (16). Therefore, understanding the pathogen spectrum, Antimicrobial Resistance (AMR) patterns, and associated risk factors for T2DM complicated by UTI is crucial for optimizing clinical management strategies. The findings of this study are generally consistent with those of recent studies (17, 18). Among the distribution of pathogens in T2DM patients with UTIs, Gram-negative bacteria account for the highest proportion, mainly *Escherichia coli* and *Klebsiella pneumoniae*. *Enterococcus faecalis* is the main Gram-positive bacteria. *Candida glabrata*, *Candida albicans*, *Enterococcus faecalis*, and *Enterococcus faecium* are also pathogens with high isolation rates. This difference may be related to the clinical characteristics of the selected research subjects and the differences in the composition of colonizing bacteria in the infection acquisition environment (19). This study shows that *Escherichia coli* in Gram-negative bacteria is the main pathogen causing UTIs complicated with T2DM. The reasons involve the colonization of *Escherichia coli* in and around the urethra, ascending infection of the bladder along the urethra, and invading and replicating the uroepithelium through biofilms. It also colonizes the kidneys, causing tissue damage and increasing the risk of bacteremia (20).

Currently, the global trend of AMR is increasingly severe, posing a significant threat to the treatment of infectious diseases. As resistance continues to rise, treatment options are becoming increasingly limited, particularly in the context of the annual increase in resistance to fluoroquinolone and cephalosporin antibiotics. The antimicrobial resistance data from this study are therefore particularly important. Antibiotic susceptibility analysis showed that *Escherichia coli* resistance rates to levofloxacin, ampicillin, and cefuroxime were all greater than 50%. This indicates that its resistance to common antibiotics has reached a high level, particularly in diabetic women with UTIs, where the risk of multidrug resistance is higher, further highlighting the challenges in clinical treatment (21). Consistent with the findings of this study, resistance of *Escherichia coli* to common antibiotics has significantly increased in recent years, especially to fluoroquinolone drugs (22). The reasons for this may include abnormal urinary tract structure and inappropriate use of antibiotics in clinical practice (12), or the spread of *Escherichia coli* resistance genes and its intrinsic defense mechanisms (23). The good susceptibility of *Escherichia coli* to tigecycline and amikacin observed in this study provides an important reference for empirical treatment. Tigecycline, as a broad-spectrum antibiotic, can effectively combat various resistant strains. Especially as fluoroquinolone resistance rises significantly, tigecycline can serve as an alternative treatment option. Amikacin, an aminoglycoside antibiotic, typically demonstrates strong antibacterial activity against multidrug-resistant bacteria, particularly Gram-negative bacteria. Studies show that fluconazole resistance in *Candida* species is significantly increasing in blood cultures and is more prominent in diabetic patients (24). The pathogen distribution and AMR patterns revealed in this study provide epidemiological data for empirical antibiotic treatment of T2DM complicated by UTI, suggesting that clinicians should prioritize coverage of Gram-negative bacteria in empirical therapy and tailor personalized treatment plans through rapid and accurate pathogen detection and antibiotic susceptibility testing.

### 4.2 The diagnostic value of PCT

PCT is a glycoprotein composed of 116 amino acids, secreted and released by thyroid C cells (25). In healthy individuals, serum PCT concentrations are extremely low. However, during inflammation, particularly in the context of bacterial infections, various tissues and cells throughout the body can produce and release PCT into the bloodstream. Therefore, serum PCT levels serve as a sensitive indicator of the severity of bacterial infections and play a crucial role in the diagnosis, monitoring, and clinical decision-making related to infectious diseases (26). Schuetz et al.’s Cochrane systematic review on PCT-guided antibiotic use in acute respiratory infections, although studying different subjects, provides high-quality evidence for the sensitivity and specificity of PCT as a bacterial infection marker. Relevant studies have shown that PCT has significant predictive potential in bacterial Urinary Tract Infections (27, 28). Research has found that PCT has good diagnostic characteristics in diagnosing diabetic foot infections, particularly with potential value in differentiating between soft tissue infection and osteomyelitis (29). The clinical application of PCT is not limited to the diagnosis of infection, but can also be used to monitor the treatment response of infection. During antibiotic treatment, a decrease in PCT levels usually indicates that the infection is under control, while a persistently high level of PCT suggests that there may be treatment failure or persistence of the infection (30). In this study, serum PCT levels were found to be significantly elevated in the infection group compared to the T2DM group, with a statistically significant difference (P< 0.05). ROC curve analysis showed that PCT has a moderate value in the diagnosis of T2DM complicated by UTIs, with an area under the curve (AUC) of 0.700 (95% CI, 0.628-0.772). This indicates that PCT can serve as an auxiliary diagnostic biomarker for T2DM complicated by UTIs, helping clinicians to promptly determine whether a patient has an infection.

### 4.3 Clinical data comparisons

In the results of this study, the comparison of general characteristics showed statistically significant differences in gender, hospitalization days, and WBC between the Infection group and the T2DM group. Multivariate logistic regression confirmed that female, prolonged hospitalization, elevated WBC, and elevated PCT were independent risk factors for T2DM complicated by UTIs. The proportion of UTIs in female patients was relatively high, which may be related to the special physiological structure of women. The female urethra is short and straight, and the lower estrogen levels and changes in the urinary tract microbiome after menopause in women are more susceptible to retrograde bacterial infection (31). In a case-control study, a statistically significant association was observed between hospitalization duration ≥ 14 days and the occurrence of catheter-associated UTIs. Multivariate regression analysis showed that even after controlling for potential confounding variables, prolonged hospitalization remained an independent risk factor for  catheter-associated UTIs, with an adjusted OR of 16.168 (10.232, 25.546) (32). Studies have shown that in patients with diabetes complicated with UTIs, sepsis is significantly positively correlated with serum WBC count. The combined analysis of C-reactive protein, serum WBC count, and albumin test results, can provide an early identification basis for sepsis in patients with diabetes complicated with UTIs (33). Age, ethnicity, marital status, smoking, alcohol consumption, duration of diabetes, presence of hypertension, BMI, fasting plasma glucose, and HbA1c showed no statistically significant differences, indicating that these factors had no significant impact on the occurrence of UTIs in this study.

### 4.4 Limitations

It is noteworthy that, in addition to the aforementioned risk factors, medications (primarily SGLT2 inhibitors), glycemic control, coexisting conditions (such as diabetic nephropathy and a history of hypertension), and disease severity may also influence the risk of UTIs complicated with T2DM (34). However, this study did not conduct an in-depth analysis of these factors, which is a deficiency. We plan to supplement this in future research. Although retrospective case-control studies are practical for quickly obtaining data and can provide preliminary clues for observational studies, retrospective studies often cannot adequately control for all potential confounding factors and may be subject to selection bias and recall bias. Future research considering prospective cohort studies may help overcome these limitations and further validate and refine these research conclusions. The patients in this study were all from a single hospital, which may limit the general applicability and external validity of the findings. Patients from different regions and populations may have different risk factors and treatment regimens. Therefore, the results of this study may not fully represent a broader population of patients with T2DM. We acknowledge this limitation and plan to further explore relevant factors in different regions and patient populations in future multi-center studies.

## 5 Conclusion

This study focused on the pathogen distribution, antibiotic resistance, and diagnostic efficacy of serum PCT in T2DM complicated by UTIs. It revealed the unique pathogen distribution in these patients, predominantly Gram-negative bacteria, and demonstrated significant resistance to common antimicrobial agents, highlighting the severe challenge of current AMR. These findings emphasize the importance of performing urine culture and antibiotic susceptibility testing in clinical practice for patients with T2DM complicated by UTIs to guide precise antibiotic selection and optimize empirical treatment strategies. Furthermore, serum PCT levels were significantly elevated in the Infection group and showed moderate diagnostic value for T2DM complicated by UTIs (AUC=0.700), suggesting its potential as an auxiliary biomarker for clinical decision-making, aiding in the differential diagnosis of infection status, and potentially providing a reference for disease assessment and monitoring treatment response. In summary, the results of this study provide some epidemiological and clinical evidence for optimizing the diagnosis and treatment of T2DM complicated by UTIs.

## Data Availability

The raw data supporting the conclusions of this article will be made available by the authors, without undue reservation.
